# Dual Anti-Glomerular Basement Membrane and Anti-Neutrophil Cytoplasmic Antibodies—Positive Rapidly Progressive Glomerulonephritis with Rheumatoid Arthritis and Sjogren’s Syndrome: A Case Report and Literature Review

**DOI:** 10.3390/jcm11226793

**Published:** 2022-11-16

**Authors:** Ting Cheng, Huiwen Zhi, Yunxiao Liu, Shengxiao Zhang, Ziyi Song, Yafeng Li

**Affiliations:** 1 Department of Rheumatology, The Second Hospital of Shanxi Medical University, Taiyuan 030000, China; 2 Key Laboratory of Cellular Physiology, Shanxi Medical University, Ministry of Education, Taiyuan 030000, China; 3 Department of Nephrology, Shanxi Provincial People’s Hospital (Fifth Hospital), Shanxi Medical University, Taiyuan 030000, China; 4 Department of Pathology, Shanxi Provincial People’s Hospital (Fifth Hospital), Shanxi Medical University, Taiyuan 030000, China; 5 Shanxi Provincial Key Laboratory of Kidney Disease, Taiyuan 030000, China; 6 Core Laboratory, Shanxi Provincial People’s Hospital (Fifth Hospital), Shanxi Medical University, Taiyuan 030000, China; 7 Academy of Microbial Ecology, Shanxi Medical University, Taiyuan 030000, China

**Keywords:** rapidly progressive glomerulonephritis, anti-glomerular basement membrane antibody, perinuclear anti-neutrophil cytoplasmic antibody, rheumatoid arthritis, Sjogren’s syndrome

## Abstract

Rapidly progressive glomerulonephritis (RPGN) is a life-threatening disease characterized by rapid progressive deterioration of renal function and extensive formation of crescents. Some antibodies tend to be positive, such as a perinuclear anti-neutrophil cytoplasmic antibody (p-ANCA) and anti-glomerular basement membrane (anti-GBM) antibodies, in most patients with the disease. However, cases of double positivity for the above antibodies are considered to be rare. In addition, both rheumatoid arthritis (RA) and Sjogren’s syndrome (SS) are deemed to be independent immune disorders that can cause renal impairment. Nevertheless, the association between RPGN and these two diseases has not been elucidated in previous studies. Here, we provide a case of RPGN with the concurrence of RA and SS characterized by double positivity in anti-GBM antibodies and p-ANCA. After aggressive treatment with cyclophosphamide, glucocorticoids, and plasma exchange, the patient improved significantly. Despite the malignant event of arteriovenous fistula rupture and bleeding during treatment, the patient survived with renal function recovery for the rest of the follow-up period.

## 1. Introduction

Rapidly progressive glomerulonephritis (RPGN) is characterized by the rapid deterioration of renal function and the formation of crescents in a short period.

PRGNS are generally considered to be of two types. The former is characterized by renal injury, pulmonary hemorrhage, and a positive anti-GBM antibody, which is the so-called Goodpasture’s syndrome. The latter is autoantibodies to neutrophil cytoplasmic antigens (ANCA) and clinicopathological traits of small vessel vasculitis. However, double positivity for anti-GBM and ANCA is thought to be relatively rare.

Rheumatoid arthritis (RA) and Sjogren’s syndrome (SS) are both autoimmune diseases that can affect the whole body, and there were a few cases of kidney involvement in the previous reports. Mesangial glomerulonephritis is the most common histological lesion of RA renal injury [1]. Furthermore, renal involvement in SS consists principally of tubulointerstitial nephritis [2]. The association of crescentic glomerulonephritis is unfamiliar, whether with RA or SS. The co-occurrence of RA, SS, and RPGN has yet to be reported. Here, we report a case of double antibody positive, including ANCA and anti-GBM antibodies, complicated by RA, SS, and PRGN in an elderly female. We compared this case with those with a single positive anti-GBM and single positive anti-ANCA and summarized the clinical features, diagnosis, management, prognosis, and the likelihood of recurrence.

## 2. Case Presentation

A 62-year-old female was admitted to the nephrology department of our hospital on 4 August 2021, because of a progressive increase in serum creatinine for a short period. Her renal function deteriorated dramatically, as indicated by a fourfold increase (from 119 to 440 μmol/L) in serum creatinine and a twofold increase (from 12.49 to 24.4 mmol/L) in urea nitrogen within two months. At the same time, there was a marked decrease in her urine output accompanied by hematuria, proteinuria, and hypoalbuminemia. Since the onset of the disease, the patient complained of nausea, fatigue, poor appetite, prolonged stiffness, and significant weight loss. Fever, hemoptysis, diarrhea, oliguria, and edema were not seen during the course.

In 2019, a local hospital diagnosed the patient with concurrent Sjogren’s syndrome and rheumatoid arthritis. Some oral medications, such as prednisone acetate tablets and hydroxychloroquine, are prescribed to control the condition. The fact that antinuclear antibodies and rheumatoid factor levels continue to rise suggests that these interventions are not working well. In May 2021, she was diagnosed with dilated cardiomyopathy (NYHA Class III) with paroxysmal tachycardia after experiencing chest tightness and shortness of breath. To make matters worse, she had abnormal renal and liver function with microcytic hypochromic anemia. Based on the multi-organ diseases mentioned above, corresponding evidence-based interventions such as diuresis, cardiac function improvement, and anemia correction were given. No information was available on related similar genetic disorders.

On admission to our hospital, the creatinine level rose to 520.06 mmol/L, and urea nitrogen levels increased to 26.33 mmol/L. Physical examination found pulse 70 ts/minute, blood pressure 121/79 mmHg, and pale skin. There were multiple enlarged nodules in the proximal interphalangeal joints of both hands.

Other laboratory data included the following values: serum albumin of 29.34 and uric acid of 556.50 μmol/L. Urinalysis showed urinary protein 3+, and the number of red blood cells was full in the field of vision. (The number of deformed red blood cells accounted for 65 percent. The deformed red blood cells included shadow red, ring, and double circle). Blood routine showed hemoglobin 74 g/L. The Erythrocyte sedimentation rate was 50 mm/h (0–15 mm/h). C-reactive protein was 8.67 mg/L (0–5 mg/L). Serological tests were positive for antinuclear antibodies (titer 1:100) and anti-GBM antibodies (not quantified). Perinuclear-ANCAs (P-ANCA) were detected in the serum, with specificity for myeloperoxidase (3+). Antinuclear antibody fluorescence assay was as follows: rheumatoid factor (50.10 IU/mL); anti-Sjogren syndrome A antibody (2.49 AI); anti-Sjogren syndrome B antibody (4.38 AI). Plasma complement (C) 3 was slightly decreased at 0.78 g/L (normal range 0.90–1.8 g/L), whereas C4 was regular at 0.17 g/L (0.1–0.4 g/L). Both IgG and IgA were above the upper limit of the normal range: respectively 39.60 g/L (7–16 g/L) and 6.53 g/L (0.7–4 g/L), whereas IgM was within the normal range:1.60 g/L (0.4–2.3 g/L). Immunoglobulin KAPPA was 10.10 g/L (1.7–3.7 g/L), and Immunoglobulin LAMBDA was 4.55 g/L (0.9–2.1 g/L).

**Figure 1:**
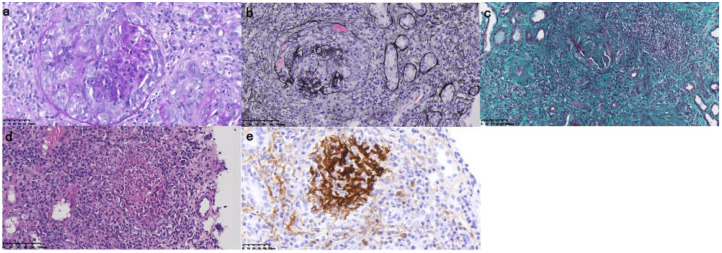
The pathologic features of renal biopsy. Cellular crescent formations of the glomeruli were observed with periodic acid-Schiff (PAS) staining in light microscopy ((**a**); 400×). Neutrophil infiltration and diffuse interstitial fibrosis in the interstitium, as well as focal tubular atrophy, were found with periodic acid-silver methenamine (PASM) and Masson staining ((**b**,**c**); 400×). Focal neutrophil infiltration and small abscess formation were revealed by hematoxylin-eosin (HE) staining ((**d**); 400×). Immunohistochemical staining showed IgG3 deposited along capillary walls ((**e**); 400×).

A renal biopsy was performed on 6 August 2021. Light microscopy revealed 19 glomeruli, of which 13 were segmentally sclerosed, 13 were crescent-shaped, 11 were cellular, and 2 were fibrocystic (Figure 1a). The glomerular tangential volume was larger (maximum diameter: 165–275μm). Mesangial cells and stroma were proliferating, and capillary loops were not patented well. Some glomeruli were infiltrated with 10–20 neutrophils. The renal tubule interstitium showed severe chronic and moderate acute changes, interstitial fibrosis ++, focal tubular atrophy (Figure 1b,c), focal neutrophil infiltration, and small abscess formation (Figure 1d). No apparent lesion was observed in arterioles. The renal light microscopy findings were consistent with crescentic glomerulonephritis. Direct immunofluorescence revealed linear deposition of IgG (2+) and Fibrin (±) in the mesangial region and the vascular loops. The thread-like deposition positivity raised the possibility of anti-GBM glomerulonephritis (GN). This renal immunohistochemistry showed IgG (+) and IgG3(vascular loops 2+), indicating the diagnosis of anti-GAM GN was accurate (Figure 1e). Based on the renal needle biopsy findings and rapidly deteriorating renal function, rapidly progressive glomerulonephritis was diagnosed.

Given the positivity of the double antibody and the formation of a pathologically massive crescent, very high doses of hormone pulse therapy and plasma exchange are necessary. The patient was treated with a 500 mg i.v. pulse of methylprednisolone sodium succinate (pulse therapy) for three consecutive days at a time, twice in total (10–12 August August; 20–22 August), and was then sustained on 40 mg daily. Then, she received IV cyclophosphamide 400 mg daily, during which plasmapheresis was performed ten times and bedside hemofiltration nine times.

After aggressive treatment, the patient’s condition became stable. Additionally, the laboratory parameters showed BUN 23.19 mmol/L, Cr 244.4 μmol/L, serum albumin 36.62 g/L, MPO++, PR3-, GBM-, pANCA-, cANCA-.

During the treatment, the patient felt discomfort in the lower abdomen. Color Doppler ultrasound of the urinary system showed that a large blood clot in the right ureter and bladder leads to a plump right kidney with diffuse lesions indicative of right hydronephrosis. Simultaneously, an area of scattered blood flow signals was noted in the lower part of the right kidney, which indicated the development of a complication of a renal arteriovenous fistula. To prevent bleeding from rupture of the renal arteriovenous fistula, the patient was instructed to rest in bed, and continuous bladder irrigation was given. Considering the potential risk of renal injury caused by interventional therapy and contrast agent, supportive conservative treatment was implemented instead of interventional therapy. During the next year of follow-up, the patient’s condition was stable, and no other malignant events occurred.

## 3. Discussion

RPGN is a life-threatening disease characterized by rapid progressive deterioration of renal function clinically and extensive crescentic formation pathologically. Three patterns can be distinguished by immunofluorescence in histology, and the pathogenesis varies. First, there is linear immunofluorescence because of antibodies against the GBM. Then, there is a granular deposition of circulating or in situ immune complexes. Finally, few immunoglobulins can be found, and serological identification shows positive ANCA. Furthermore, there is an overlap between different phenotypes in patients, as some may be positive for both ANCA and anti-GBM. Three RPGN patients with both MPO-ANCAs and anti-GBM antibodies were reported in 1989 for the first time [3], and such cases have been covered worldwide over the subsequent years [4,5]. A study showed that in patients with the anti-GBM disease, the detection rate of double positivity for ANCA and anti-GBM reached up to 30%, particularly in middle-aged females [6]. Double positivity for ANCA and anti-GBM was reported to lead to a similar or worse outcome of renal vasculitis compared with just single positivity or none [5,7].

In the present case, we describe a patient diagnosed with RA and SS at the same time two years ago and subsequently developed rapidly progressive glomerulonephritis with coexistent p-ANCA and anti-GBM antibodies. RA is a systemic disorder that principally involves joints. As we all know, RA patients may develop various extraarticular symptoms that are usually relevant to immune-complex mediated vasculitis. Renal involvement in RA is relatively rare, and the most frequent histological lesion is mesangial glomerulonephritis, followed by minimal change nephrotic syndrome and p-ANCA-positive crescentic glomerulonephritis [1]. SS is a chronic autoimmune disease characterized by lymphocyte infiltration of exocrine glands, leading to multiple systems and organ disorders. The renal injury affects 0.3–27.0% of patients. Tubulointerstitial nephritis represents the most common form of nephropathy in SS, with clinical manifestations such as renal tubular acidosis, diabetes insipidus, Gitelman syndrome, or Fanconi syndrome [8]. Glomerular involvement is less frequent in SS patients. The renal prognosis is often favorable in patients with SS, whereas renal failure may occur in rare cases.

The patient was not tested for ANCA and anti-GBM antibodies at the time of diagnosis of RA and SS two years ago in the Second Hospital of Shanxi Medical University. Later serological identification showed positive MPO and negative GBM in May in the First Hospital of Shanxi Medical University, though the renal function was within normal limits. MPO is the main target antigen of ANCAs, which are autoantibodies specific for antigens located in the cytoplasmic components of neutrophils and monocytes and generally tested in autoimmunity diseases such as ANCA-associated vasculitides (AAV), featured by necrotizing vasculitis typically involving small vessels such as renal arteriole [9]. Therefore, the patient was possibly ANCA positive at that time or earlier. 

In recent years, ANCA has been detected in patients with connective tissue diseases such as RA and SS. It has been suggested that ANCA is generated due to the destruction of neutrophils in the synovial tissue in patients with RA during inflammation [10]. The positive rate of ANCA in RA patients ranged from 16% to 52% [11]. In patients with early RA, not only are pANCAs statistically associated with specific serological markers of RA, such as rheumatoid factor, antiperinuclear factor, and antikeratin antibodies but they also predict rapid radiographic joint destruction [12]. P-ANCAs in RA signify severe disease with a pro-inflammatory response, and there is a powerful and independent association between ANCA and RA-related nephropathy [13]. Draibe J et al. reported that 33 of 35 patients developed AAV years after the diagnosis of RA [14]. Three patients reported by Kurita N et al. Were diagnosed with RA and developed AAV 7–10 years later [10]. Moreover, the presence of ANCA in SS patients is less common. Ramos-Casals et al. studied 402 patients with primary SS, and only 13 patients (3.2%) had positive ANCA [15]. Furthermore, in previous research of ANCA-positive primary SS patients, a higher prevalence of extra exocrine gland symptoms, such as pulmonary, renal, and neurological involvement, was evident [2,15]. SS patients with p-ANCA showed a lower cumulative end-stage renal disease (ESRD)-free survival rate than those without [16]. Guellec D et al. reported that 22 AAV patients complicated with SS were diagnosed with SS first and AAV later [17]. Therefore, ANCA should be routinely tested for common connective tissue diseases to avoid AAV, RPGN, or even more severe consequences. 

Combining cyclophosphamide with glucocorticoid and plasmapheresis is the standard therapy for RPGN. Therefore, these regimens were applied to the patient. She improved significantly in symptoms and renal function. The long-term prognosis after remission is still uncertain, and later examinations should be followed, which is also a limitation of this case.

ANCA may be positive in RA or SS patients, and persistent positive ANCA may lead to RPGN, a life-threatening disease. In addition, p-ANCAs in RA or SS may signify severe disease with a pro-inflammatory response, including more obvious joint destruction and a higher prevalence of extra exocrine gland symptoms. Therefore, multiple antibody screening, excluding disease-specific antibodies, is worthwhile for the prevention or early diagnosis of potentially serious complications.

## Data Availability

Not applicable.

## References

[1] Icardi A., Araghi P., Ciabattoni M., Romano U., Lazzarini P., Bianchi G. (2003). Kidney involvement in rheumatoid arthritis. Reumatismo.

[2] Wang W.J., Wu H.S., Chu T.S. (2011). Anti-neutrophil Cytoplasmic Antibody-associated Pauci-immune Crescentic Glomerulonephritis Complicating Sjogren’s Syndrome. J. Formos. Med. Assoc..

[3] O’Donoghue D.J., Short C.D., Brenchley P.E.C., Lawler W., Ballardie F.W. (1989). Sequential development of systemic vasculitis with anti-neutrophil cytoplasmic antibodies complicating anti-glomerular basement membrane disease. Clin. Nephrol..

[4] Rutgers A., Slot M., Paassen P.V., Vriesman P.V.B., Heeringa P., Tervaert J.W.C. (2005). Coexistence of Anti-Glomerular Basement Membrane Antibodies and Myeloperoxidase-ANCAs in Crescentic Glomerulonephritis. Am. J. Kidney Dis..

[5] Mcadoo S.P., Tanna A., Hrušková Z., Holm L., Weiner M., Arulkumaran N., Kang A., Satrapová V., Levy J., Ohlsson S. (2017). Patients double-seropositive for ANCA and anti-GBM antibodies have varied renal survival, frequency of relapse, and outcomes compared to single-seropositive patients. Kidney Int..

[6] Zhao J., Yang R., Cui Z., Chen M., Zhao M.-H., Wang H.-Y. (2007). Characteristics and outcome of Chinese patients with both antineutrophil cytoplasmic antibody and anti-glomerular basement membrane antibodies. Nephron Clin. Pract..

[7] Balderia P.G., Andeen N., Jefferson J.A. (2019). Characteristics and Outcomes of Patients with Anti-Glomerular Basement Membrane Antibody Disease and Anti-Neutrophil Cytoplasmic Antibodies. Curr. Rheumatol. Rev..

[8] François H., Mariette X. (2020). Renal involvement in Sjögren’s syndrome. Nephrol. Ther..

[9] Sebastiani M., Luppi F., Sambataro G., Villegas D.C., Cerri S., Tomietto P., Cassone G., Bocchino M., Atienza-Mateo B., Cameli P. (2021). Interstitial Lung Disease and Anti-Myeloperoxidase Antibodies: Not a Simple Association. J. Clin. Med..

[10] Kurita N., Mise N., Fujii A., Mori M., Sai K., Nishi T., Suzuki T., Tagawa H., Sugimoto Y. (2010). Myeloperoxidase-antineutrophil cytoplasmic antibody-associated crescentic glomerulonephritis with rheumatoid arthritis: A comparison of patients without rheumatoid arthritis. Clin. Exp. Nephrol..

[11] Spoerl D., Pers Y.M., Jorgensen C. (2012). Anti-neutrophil cytoplasmic antibodies in rheumatoid arthritis: Two case reports and review of the literature. Allergy Asthma Clin. Immunol. Off. J. Can. Soc. Allergy Clin. Immunol..

[12] Mustila A., Paimela L., Leirisalo-Repo M., Huhtala H., Miettinen A. (2000). Antineutrophil cytoplasmic antibodies in patients with early rheumatoid arthritis: An early marker of progressive erosive disease. Arthritis Rheum. Off. J. Am. Coll. Rheumatol..

[13] Mustila A., Korpel M., Mustonen J., Helin H., Huhtala H., Soppi E., Pasternack A., Miettinen A. (1997). Perinuclear antineutrophil cytoplasmic antibody in rheumatoid arthritis. A marker of severe disease with associated nephropathy. Arthritis Rheum. Off. J. Am. Coll. Rheumatol..

[14] Draibe J., Salama A.D. (2015). Association of ANCA associated vasculitis and rheumatoid arthritis: A lesser recognized overlap syndrome. SpringerPlus.

[15] Ramos-Casals M., Nardi N., Brito-Zerón P., Aguiló S., Gil V., Delgado G., Bové A., Font J. (2006). Atypical Autoantibodies in Patients with Primary Sjogren Syndrome: Clinical Characteristics and Follow-Up of 82 Cases. Semin. Arthritis Rheum..

[16] Lee S.B., Choi H., Kim M.K., Jung S.M., Song J.J., Park Y.-B., Lee S.-W. (2019). Can antineutrophil cytoplasmic antibody positivity at diagnosis predict the poor outcomes of Sjogren’s syndrome?. Rheumatol. Int..

[17] Guellec D., Gall C.L., Groh M., Hachulla E., Karrase A., Charles P., Dunogué B., Abad S., Alvarez F., Gérard F. (2015). ANCA-associated vasculitis in patients with primary Sjogren’s syndrome: Detailed analysis of 7 new cases and systematic literature review. Autoimmun. Rev..

